# Dentin Exposure after Tooth Preparation for Laminate Veneers: A Microscopical Analysis to Evaluate the Influence of Operators’ Expertise

**DOI:** 10.3390/ma15051763

**Published:** 2022-02-26

**Authors:** Roberto Sorrentino, Gennaro Ruggiero, Bruna Borelli, Alberta Barlattani, Fernando Zarone

**Affiliations:** 1 Department of Neurosciences, Reproductive and Odontostomatological Sciences, Division of Prosthodontics and Digital Dentistry, University “Federico II” of Naples, 80131 Naples, Italy; roberto.sorrentino@unina.it (R.S.); unabellabruna@libero.it (B.B.); zarone@unina.it (F.Z.); 2 Department of Clinical Sciences and Translational Medicine, Tor Vergata University, 00133 Rome, Italy; barlattanialberta@gmail.com

**Keywords:** ceramics, porcelain, veneers, minimally invasive, dentin, cad/cam, tooth preparation, esthetic dentistry, laminate veneers, veneer preparation

## Abstract

Background: To assess the quantity of dentin exposure detected by 3 operators with different clinical expertise for 2 designs of tooth preparation for laminate veneers: window (WI) and butt joint (BJ). Methods: 20 intact maxillary central incisors were collected and then prepared for laminate veneers to a depth of 0.6 mm, with a cervical mini-chamfer finish line of 0.3 mm. Each prepared tooth was analyzed by 3 operators with different expertise: undergraduate student (ST), general practitioner (GP), and prosthodontist (PR), at sight under magnification. Besides descriptive statistics (CI 95%), 2-way ANOVA and Games–Howell tests were used to analyze differences among groups (α = 0.05). Results: The means of percentage and area of detected dentin exposure were WI = 30.48%, 21.57 mm^2^; BJ = 30.99%, 21.97 mm^2^; ST/WI = 22.82%, 16.44 mm^2^; GP/WI = 58.05%, 40.64 mm^2^; PR/WI = 10.55%, 7.63 mm^2^; ST/BJ = 28.99%, 20.83 mm^2^; GP/BJ = 40.56%, 28.32 mm^2^; PR/BJ = 23.42%, 16.75 mm^2^. Significant differences were found between ST/WI vs. GP/WI (*p* = 0.005) and GP/WI vs. PR/WI (*p* < 0.001). Conclusions: There was no difference in detection of exposed dentin among operators with different expertise for BJ preparation, whereas differences were found between the general practitioner and the other 2 operators in WI. Moreover, the quantity of exposed dentin was not related to different tooth preparation designs.

## 1. Introduction

In recent decades, laminate veneers (LVs) have become a widespread treatment option for the possibility of meeting the demand for long-lasting, highly aesthetic, and minimally invasive restorations [1,2,3].

An LV is defined as a superficial or attractive display in multiple layers that restores a tooth at the incisal, buccal, and/or part of palatal and interproximal surfaces [4]. It could be made of porcelain-based or porcelain-free materials, such as feldspar ceramics, lithium disilicate, zirconia, or zirconia-reinforced lithium silicate [5,6,7]. This type of restoration allows for the restoring of dental aesthetics, in cases of misalignment, wear, discoloration, fractures, and morphological alterations [8,9]. Moreover, additional partial veneers, “minimal preparation” and even “no-preparation (or prepless)” laminate veneers can be used in situations involving a minimum ceramic application, with a thickness of 0.3–0.5 mm, such as in the closure of diastemas, limited reshaping of front teeth, treatment of microcracks, enamel defects, and minor discolorations [9,10,11].

With LVs, the teeth are considerably preserved, thanks to the reduced thickness required by the new biomimetic ceramic materials and by the efficient bond with enamel [1]. The survival rate of LVs is negatively affected by veneer preparations extending into dentin, allowing enamel an optimal adhesion [1,2,3,12,13,14].

**Figure 1:**
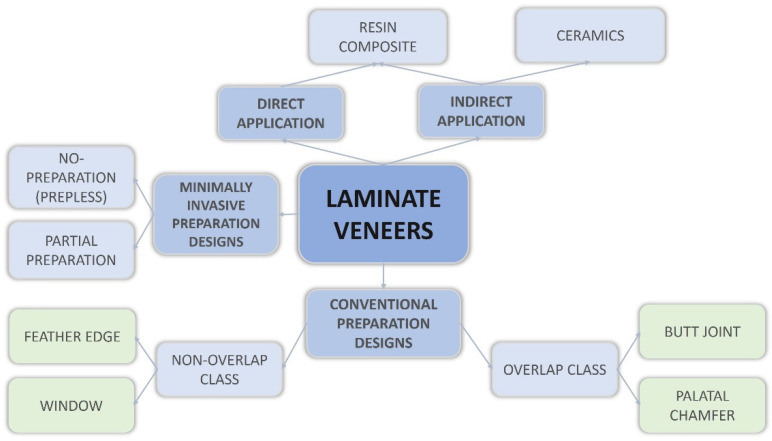
Diagram showing all types of laminate veneers.

Four typologies of tooth preparation designs are mostly used for LVs: window (WI), feather edge, butt joint (BJ) (“incisal bevel”), and palatal chamfer (“overlapped”) [15]. The first two configure the “non-overlap” class, the other two the “overlap” class (Figure 1) [16,17].

Besides, high survival rates were reported in the literature for the different preparation designs [17]. The incisal-covered preparation designs for LVs show an increased risk of failure compared to those without incisal coverage [18]. Among incisal-covered designs, the BJ is a type of preparation that affects the tooth strength less than the palatal chamfer, while the latter is more prone to ceramic fractures [16,19]. It is worth noting that the location of LVs is also a relevant factor in the risk of failure. Particularly, maxillary central and lateral incisors prepared with a palatal chamfer exhibited greater fracture strength than those prepared with a WI preparation [18,20]. Conversely, maxillary canines prepared with the WI design were more resistant to fracture than those prepared with a palatal chamfer [20]; however, it has to be pointed out that the preparation of designs with an incisal covering yield better aesthetic results than designs without incisal coverage [2].

In the present study, the BJ and WI geometries were selected as the most scientifically validated and clinically used preparation designs for laminate veneers [16,18]. Given that the thickness of enamel is not homogenous and can vary both in the mesio-distal and apico-coronal directions [21], designs limited to the buccal surface (i.e., window) are somehow more conservative than preparations involving the incisal margin reduction (i.e., feathered, butt joint, palatal chamfer) [2]; therefore, the more dental tissue is removed, the greater the risk of exposing dentin [22,23].

Moreover, several clinical investigations proved that different operative approaches could significantly affect dentin exposure; nevertheless, a correlation between particular preparation designs and the amount of exposed dentin has not yet been defined [22,23]; therefore, freehand preparation was chosen in the present study to simulate the worst clinical scenario.

In any case, detecting the exposition of dentin structure during tooth preparation is not easy, affecting the effectiveness of bonding. In this regard, optical magnification systems can be helpful to better visualize dental tissues [24]. Furthermore, there could be inter- and intra-individual variability in the recognition of tooth hard tissues and, therefore, of exposed dentin. In particular, the inter-individual variability could be relevant in the case of operators with different clinical expertise.

To date, to the authors’ knowledge, there are no investigations evaluating possible differences in the identification of hard tissues in prepared teeth with different preparation designs, using magnification systems, between operators with different clinical expertise.

The purpose of the present in vitro investigation was to assess the areas of dentin exposure with the use of a stereomicroscope in 2 different designs of tooth preparations (WI and BJ) for LVs.

For this purpose, two null hypotheses were formulated:

1. there is no association among different designs of tooth preparation for LVs and the amount of dentin exposure;
2. there is no difference both intra- and inter-individual in the discrimination under magnification between prepared enamel and dentin for operators with different clinical expertise.

## 2. Materials and Methods

### 2.1. Specimen Selection

**Figure 2:**
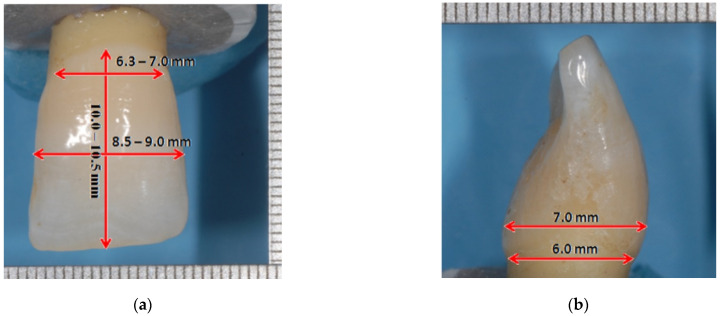
Reference scale and selected anatomical dimensions according to the parameters described by Nelson and Ash. (**a**) crown length and mesiodistal diameters; (**b**) buccopalatal diameters. Each dash is spaced half a millimeter apart.

Twenty maxillary central incisors extracted for periodontal pathologies, free of caries and restorations, were collected among the discarded teeth extracted at the Department of Oral Surgery of the University Hospital “Federico II” of Naples. Also, teeth with wear were excluded. Any plaque, calculus, and periodontal ligament residues were removed using ultrasonic instruments, curettes, and silicone rubber polishers. The collected teeth were extracted from patients with an age range of 35 to 50 years. The maxillary central incisors were included in the study if fulfilling the anatomical parameters described by Nelson and Ash [25] (Figure 2):

- crown length: 10–10.5 mm;
- root length: 12–13 mm;
- mesiodistal diameter of crown: 8.5–9 mm;
- mesiodistal diameter of crown at cervix: 6.3–7 mm;
- buccopalatal diameter of crown: 7 mm;
- buccopalatal diameter of crown at cervix: 6 mm [25].

Therefore, all the selected specimens presented a total length of 22 ± 1 mm, buccopalatal and mesiodistal crown lengths of 7 ± 1 mm and 9 ± 1 mm, respectively.

Only the average dimensions of sample teeth but not the variation in enamel thickness were considered, so as to simulate a real clinical scenario, in which enamel thickness can vary according to the anatomy of each tooth.

The specimens were stored in a 1% solution of thymol at 25 °C immediately after extraction and for a maximum period of 4 weeks.

Subsequently, they were placed into cylinders filled with autopolymerizing resin (Orthojet Lang, Ravelli S.p.a., Milan, Italy) leaving at least 2 mm of the root exposed apically to the cemento–enamel junction, in order to make finish lines visible. The specimens were randomly numbered in ascending order to be identified individually.

### 2.2. Tooth Preparation

One experienced prosthodontist performed 2 different tooth preparation designs for LVs at sight under magnification of 16×, with a dedicated medical stereomicroscope (OPMI PROergo, Carl Zeiss AG, Oberkochen, Germany). According to the preparation designs, the specimens were divided into 2 experimental groups (*n* = 10), named WI and BJ.

For each specimen, a silicone index was made before preparation with silicone material (Platinum 85, Zhermack S.p.a., Rovigo, Italy) to check the thickness of the dental tissues removed during tooth preparation. Each silicone index was created by sectioning the index vertically through the maximum longitudinal axis of each tooth, along the buccopalatal plane. All the sectioned indexes were numbered according to the corresponding tooth.

**Figure 3:**
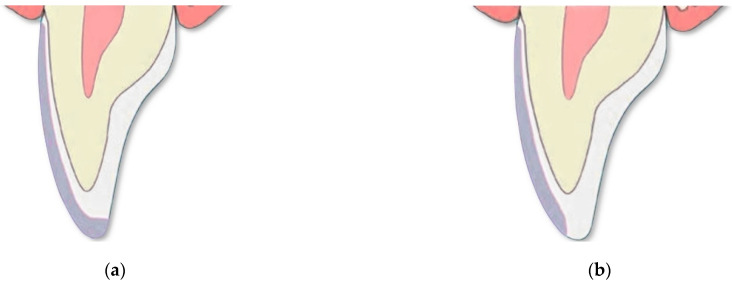
The tested preparation designs for LVs: (**a**) butt joint (BJ); (**b**) window (WI).

Each preparation was performed with dedicated diamond-coated burs (801.314.006 and 837KR.314.012, Komet, Gebr. Brasseler GmbH & Co. KG, Lemgo, Germany) mounted on a contra-angle handpiece 1:5 for micromotor (WK-99 LT, Synea, W&H Dentalwerk GmbH, Bürmoos, Austria) at 200.000 min^−1^/rpm under spray water. The specimens were prepared with a mini-chamfer cervical finish line of 0.3 mm and buccal depth of 0.6 mm. The preparation thicknesses were carefully checked with the silicone index and a millimeter-periodontal probe (Offset Williams Probe, Hu-Friedy Mfg. Co., Chicago, IL, USA), under magnification. For BJ preparations, the incisal margin was removed to a length of 2 mm (Figure 3a), while for WI preparations the incisal margin was preserved (Figure 3b). Arkansas burs (661-204-420, Komet, Gebr. Brasseler GmbH & Co. KG, Lemgo, Germany) were used for finish line polishing and surface smoothing. The polishing burs were mounted on a contra-angle handpiece 1:1 for micromotor (WK-56 LT, Synea, W&H Dentalwerk GmbH, Bürmoos, Austria), at 20.000 min^−1^/rpm under spray water.

### 2.3. Analysis of Prepared Surfaces

Immediately after tooth preparation, the dried surfaces were analyzed following a protocol similar to the one of Blunck et al. [1]. The specimens were photographed through the stereomicroscope (OPMI PROergo, Carl Zeiss AG) to better evidence enamel from dentin. A camera (Nikon F-Mount DK-10, Tokyo, Japan) was mounted on a dedicated arm of the stereomicroscope and each sample was placed onto a stable support surface, in order to obtain a repeatable and standardized focal distance. The teeth were placed so that the buccal surface was perpendicular to the optical system of the stereomicroscope.

A raster graphics editor software (Adobe Photoshop CS4 Extended v11.0, Adobe Inc., San Jose, CA, USA) was used to analyze the pictures made for each specimen. A measurement scale (1 mm = 129 px) was set referring to a known distance of the picture, displayed in Figure 2 as a reference millimeter scale.

To assess the quantity of exposed dentin, the full prepared area of the tooth was selected with the “Quick Selection Tool”, setting a diameter of 3 px and recording the number of pixels from the “Histogram” function after clicking on “click for histogram with uncached data”. This operation was made to obtain the percentage of exposed dentin area on the full prepared tooth area, according to the following formula:

% of exposed dentin area compared to the full prepared area = (exposed dentin area (px))/(prepared tooth area (px)) × 100.

In the same software, a square was drawn with a 1 mm side, according to the millimetric scale (Figure 2). Subsequently, the square was selected with the “Rectangular Marquee Tool (M)”, to obtain the number of pixels corresponding to the square area through the “Histogram” function. Finally, to know the surface area in mm^2^ of exposed dentin, the following formula was used:

Area of exposed dentin (mm^2^) = (exposed dentin area (px))/(reference square area (px)) × 1 mm^2^.

**Figure 4:**
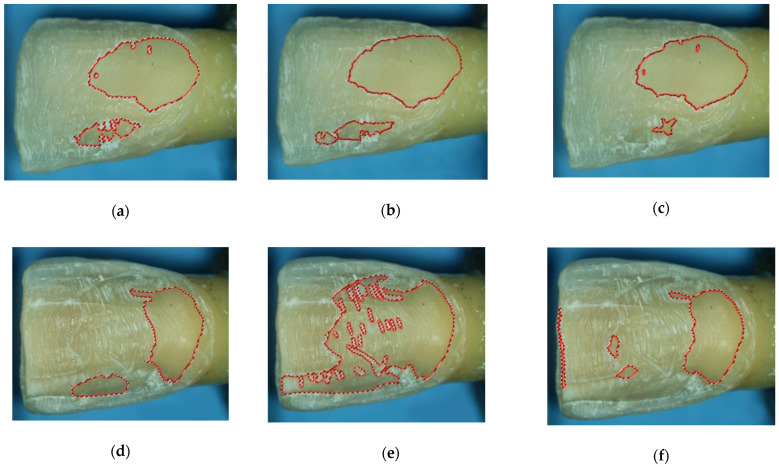
Exposed dentin surfaces were detected through digital analysis and the use of a stereomicroscope by 3 operators with different clinical expertise for 2 preparation designs. ST, undergraduate student; GP, general practitioner, PR, prosthodontist; WI, window; BJ, butt joint. (**a**) ST/WI; (**b**) GP/WI; (**c**) PR/WI; (**d**) ST/BJ; (**e**) GP/BJ; (**f**) PR/BJ. After tooth preparation, the prepared surfaces were first dried and then photographed with the stereomicroscope. A raster graphics software was used to analyze the pictures taken and to draw the perimeter of dentin tissue.

This procedure was repeated for each picture by 3 operators with different expertise: a trainee undergraduate student (ST), a general practitioner (GP), and an experienced prosthodontist (PR) (Figure 4). Operators with different clinical expertise were considered in order to report if there might be inter- and intra-individual variability in the detection of exposed dentin. Before performing the measurements, all the operators were trained to use the software with a few representative dummy pictures.

Nowadays, it is possible to accurately discriminate between enamel and dentin only through instrumental or histological investigations [26,27,28]. With regards to the operative procedures, during tooth preparation clinicians could discriminate between enamel and dentin visually, since enamel can be kept dry while dentin is characterized by a shiny appearance due to intrinsic humidity [29], and thanks to patients’ sensitivity, typically subsequent to dentin exposure if anesthesia was not injected [23]; however, to date, no clear and univocal parameters to clinically distinguish enamel and dentin have been established yet.

Although the study specimens were kept hydrated until the execution of the microscopical analysis, dehydration could occur due to environmental conditions and microscopic light; consequently, discrimination due to intrinsic moisture could not be used for the investigation.

Study operators were trained accordingly, and some specific optical and morphological features of dental tissues were considered [29]. Particularly, enamel presents with greater translucency and value, while dentin shows a more intense chroma, opacity, and more polished appearance. Also, the grooves left by burs were more pronounced and appeared like whitish stripes onto the enamel (Figure 4). Besides, the operators used the intact interproximal enamel tissue surrounding the prepared area as a reference for comparison.

With this procedure, 6 experimental groups were made: ST/WI, GP/WI, PR/WI, for the WI preparation design and ST/BJ, GP/BJ, and PR/BJ for the BJ design.

The evaluation of each operator on the prepared teeth was made by alternating the two preparation designs and observing a blue paper surface on which to rest the eyes [30], in order to minimize the impact of operators’ fatigue and avoid the related bias.

### 2.4. Statistical Analysis

The statistical analysis was performed with a statistical software program (IBM SPSS Statistics, v25; IBM Corp, New York, NY, USA) on data concerning the percentage and the mm^2^ of exposed dentin areas. Descriptive statistics (i.e., mean, standard deviation, lower- and upper-bounds with 95% confidence interval—CI) and specific tests were run to determine the overall statistical significance of the differences between the experimental groups. Particularly, the Shapiro–Wilk and the Levene tests were run, respectively, to assess the normality of the distribution of the statistical variables and to evaluate the variance homogeneity. The 2-way analysis of variance (ANOVA) was used to identify interactions among operators and preparation designs.

The Bonferroni post hoc test or the Welch test followed by the Games–Howell post hoc test were used to analyze differences among groups (α = 0.05). To consider only clinically relevant comparisons, all the possible pairwise comparisons among the 6 experimental groups were not performed; consequently, it was evaluated whether differences existed among operators within a preparation design and between preparation designs within an operator.

Moreover, a power analysis was performed with the software G*Power (v. 3.1.9.6, Universität Kiel, Germany) to determine the sample size effect. To determine the effect size, partial eta squared (η^2^) is the effect size measure for the interaction between the within and between subject variables. Approximate partial eta squared conventions are small = 0.02; medium = 0.06; large = 0.14. A medium effect size was assumed.

## 3. Results

**Figure 5:**
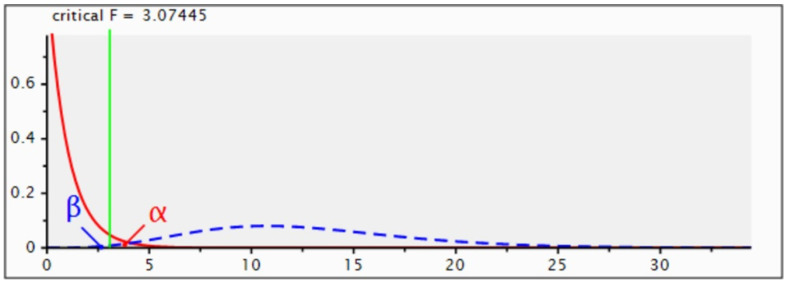
Power Analysis to define the Sample Size Effect.

There was a 99.1% of correctness in rejecting the null hypothesis of no significant effect of the interaction with 30 WI and 30 BJ measurements for a total of 60 assessments (Figure 5).

**Figure 6:**
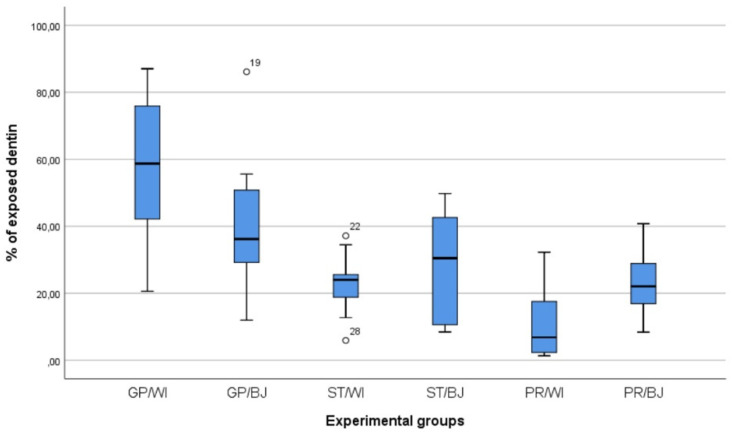
Box-plot chart showing values of the percentage of exposed dentin. Whiskers below and above box exhibit positions of minimum and maximum, whereas box spans show the first quartile to the third quartile. The median is represented by segments inside the box and suspected outliers are shown as unfilled circles. ST, undergraduate student; GP, general practitioner; PR, prosthodontist; WI, window; BJ, butt joint.

The results of the descriptive statistics about the measurements of exposed dentin (in percentage and mm^2^) are summarized in Table 1 and Table 2, while the box-plot chart of the 6 experimental groups is shown in Figure 6. The means in percentage and mm^2^ of exposed dentin for WI preparations were 30.48% and 21.57 mm^2^, while for BJ preparations were 30.99% and 21.97 mm^2^.

The 2-way ANOVA (Table 3) detected statistically significant differences between the evaluations of individual operators (ST, GP, and PR) (*p* < 0.001) but not between the 2 types of tested tooth preparation designs (WI and BJ) (*p* = 0.898). Nonetheless, the mutual interaction between the study variables showed a statistically significant difference (*p* = 0.008).

Among the operators, the Shapiro–Wilk test reported that the values were normally distributed (*p* > 0.05) for all the groups and the Levene test showed that the variances were homogeneous (*p* = 0.005).

The Bonferroni post hoc test recorded statistically significant differences between the evaluations of ST and GP (*p* < 0.001) and between GP and PR (*p* < 0.001); differently, no statistically significant difference was detected between ST and PR (*p* = 0.277) (Table 4).

Among the 6 experimental groups, the Shapiro–Wilk test reported that the values were normally distributed (*p* > 0.05) for all the groups and the Levene test showed that the variances were not homogeneous (*p* = 0.012).

Since there was a normal distribution but no homogeneity of the variances, the robust Welch test of equality of means was used and reported a significant value [*p* < 0.001 with F (5, 24.84 = 8.96)]. After the Welch test, the Games–Howell analysis was performed to evaluate whether there were any statistically significant differences between preparation designs within an operator and among operators within a preparation design. Significant differences were detected among operators within the means of the WI preparation design, particularly between ST and GP (*p* = 0.005) and between GP and PR (*p* < 0.001) (Table 4).

## 4. Discussion

LV is an efficient restorative option, offering a reliable treatment that preserves the structure of teeth while providing outstanding esthetic outcomes and patient acceptance [1,2,3,14,15].

This restorative system exhibited high longevity and low complication rates, as supported by many systematic reviews with follow-up periods ranging between 5 and 21 years, showing survival rates ranging from 87% to 96% [31,32,33,34]. Several factors affect the survival of LVs, such as cementation materials [35], quality of dental substrates (enamel vs. dentin) and mechanical properties of restorations [36], presence of previous fillings, occlusal forces, and preparation design [9,32,33,34,37].

Tooth preparation for LVs can be performed by evaluating the following aspects: buccal surface preparation (no preparation, minimal preparation, conservative, or conventional preparation), incisal preparation (overlapping or non-overlapping), proximal finish (slice or chamfer), and cervical preparation (chamfer or knife-edge) [16].

In this study, considering the different preparation designs as statistical variables and since no statistically significant differences were detected between the 2 experimental groups, WI and BJ, the first null hypothesis (no association between the 2 types of tooth preparation design and the quantity of exposed dentin) was accepted. Instead, considering the statistically significant differences detected between the 3 operators with different expertise (ST, GP, and PR), the second null hypothesis (no difference, both intra- and inter-operator) was partially rejected.

The descriptive statistics reported lower values of exposed dentin for the WI preparation than for BJ (WI = 30.48% and 21.57 mm^2^; BJ = 30.99% and 21.97 mm^2^); however, this difference was not statistically significant (*p* = 0.898), therefore, the association between the type of tooth preparation (WI and BJ) and the amount of exposed dentin was not found. It can thus be asserted that the choice between BJ and WI designs should take into consideration factors like the risk of fracture and aesthetic needs, instead of dentin exposure; therefore, the advantages and disadvantages of these preparation geometries should be considered (Table 5).

In the comparison between the means of ST, GP, and PR groups, a statistical significance was found (*p* < 0.001), in particular, between GP and the other 2 operators in the WI scenario (ST/WI-GP/WI, *p* = 0.005; GP/WI-PR/WI, *p* < 0.001). These differences show that proper training may be paramount in discriminating between prepared enamel and exposed dentin. These findings were mainly observed in the WI preparation, where the amount of dental tissue to be evaluated was wider than in the BJ preparation, with its 2-mm incisal reduction. Therefore, since the evaluated surface area was wider, then the cumulative error was higher, leading to a statistical difference only in the WI scenario.

Therefore, the recorded results show that no difference was found within the operator between preparation designs, demonstrating that, even if there is an inter-operator difference for the WI design, an intra-operator difference was not detected both in WI and BJ.

Furthermore, the mean values of exposed dentin found in the 2 different preparation designs (WI and BJ) were approximately 30%, with a 70% of exposed enamel. This value is above the minimally acceptable for the enamel exposure (40%) required to obtain an efficient bond strength [38,39].

Finally, this investigation confirms that the use of magnification devices is a useful system for discriminating between enamel and dentine and therefore to standardize the preparation procedures [1,24,40].

The present study has some limitations, mainly related to its in vitro nature. Only two preparation designs for LVs were tested, WI and BJ. The samples consisted of extracted teeth, so, clinically relevant factors related to the oral environment, in particular temperature, humidity, and optical features, were not considered; moreover, the surface analysis was two-dimensional, using images taken under magnification, so the surface shape of prepared teeth was not considered. Besides, only one operator for each level of clinical expertise was considered.

Further in vitro and in vivo investigations involving a larger sample size are needed to confirm that the various preparation designs for LVs do not determine a different amount of exposed dentin. Moreover, a larger number of operators would be advisable to confirm that there are inter-individual variabilities in the discrimination of hard tissues in prepared teeth to optimize subsequent adhesive cementation procedures [22,23].

## 5. Conclusions

Within the limitations of the present in vitro study, the following conclusions can be drawn about LVs:

1. the quantity of exposed dentin is not associated with the two considered preparation designs, namely window and butt joint;
2. the expertise of clinical operators represents a discriminating factor in identifying prepared hard dental tissues, as both an undergraduate student and an expert prosthodontist showed statistically different values from a general practitioner as to the window preparation;
3. in the butt joint preparation, no differences were found between the different operators;
4. variability was found in the inter-individual evaluation of exposed dentin following different preparation designs for LVs;
5. no intra-operator variability was detected both in window and butt joint preparations;
6. magnification tools were useful to discriminate between prepared enamel and dentin.

Further in vivo and in vitro studies would be helpful to confirm and validate the findings of the present investigation.

## Figures and Tables

**Table 1:** Results in percentage of exposed dentin areas: mean, standard deviation, and lower-upper bound (95% confidence interval).

Operators	Mean (%)	Standard Deviation	Lower-Upper Bound
ST/WI	22.82	9.23	16.21–29.42
ST/BJ	28.99	15.45	17.93–40.04
GP/WI	58.05	21.58	42.61–73.49
GP/BJ	40.56	21.25	25.35–55.76
PR/WI	10.55	10.16	3.27–17.82
PR/BJ	23.42	9.56	16.58–30.26

ST, undergraduate student; GP, general practitioner, PR, prosthodontist; WI, window; BJ, butt joint.

**Table 2:** Results in mm^2^ of exposed dentin areas: mean, standard deviation, and lower-upper bound (95% confidence interval).

Operators	Mean (mm^2^)	Standard Deviation	Lower-Upper Bound
ST/WI	16.44	7.19	11.29–21.58
ST/BJ	20.83	11.94	12.29–29.38
GP/WI	40.64	12.91	31.41–49.88
GP/BJ	28.32	14.78	17.74–38.89
PR/WI	7.63	7.86	2.01–13.25
PR/BJ	16.75	7.45	11.42–22.09

ST, undergraduate student; GP, general practitioner, PR, prosthodontist; WI, window; BJ, butt joint.

**Table 3:** Results of 2-way ANOVA.

Source	SS	df	MS	F	*p*
Corrected Model	13,694.17	5	2738.83	11.43	<0.001
Intercept	56,675.34	1	56,675.34	236.67	<0.001
Operator	11,144.83	2	5572.41	23.27	<0.001
Preparation	3.97	1	3.97	0.01	0.898
Operator × Preparation	2545.36	2	1272.68	5.31	0.008
Error	12,931.04	54	239.46		
Total	83,300.56	60			
Corrected Total	26,625.22	59			

ANOVA, analysis of variance; SS, sum of squares; df, degree of freedom (n − 1); MS, mean squares. Significant at *p* < 0.05.

**Table 4:** *p* values of post hoc comparisons.

Comparison	*p*
ST/WI-ST/BJ	0.880
GP/WI-GP/BJ	0.475
PR/WI-PR/BJ	0.083
ST/WI-GP/WI	0.005 *
ST/WI-PR/WI	0.099
GP/WI-PR/WI	<0.001 *
ST/BJ-GP/BJ	0.731
ST/BJ-PR/BJ	0.921
GP/BJ-PR/BJ	0.254
ST-GP	<0.001 *
ST-PR	0.277
GP-PR	<0.001 *

* Statistically significant differences (*p* < *0*.05). ST, undergraduate student; GP, general practitioner, PR, prosthodontist; WI, window; BJ, butt joint.

**Table 5:** Advantages and disadvantages between window and butt joint designs for laminate veneers.

Preparation Design	Advantages	Disadvantages
Window	- Decreased failure risk [18]. - Conservative [16,19]. - Does not interfere with incisal guidance [16].	- More than one path for insertion [2]. - Lower aesthetic [2,16].
Butt joint	- Better aesthetic [2,16]. - Precise seat and stop for cementation [2]. - Possibility to restore incisal guidance [2]	- Increased failure risk [18]. - Not conservative as the window design [19]. - Interfere with incisal guidance [16].

## Data Availability

The datasets used and/or analyzed during the current study are available from the corresponding author on reasonable request.
